# *Salvia deserti* Decne., an endemic and rare subshrub from Arabian desert: antidiabetic and antihyperlipidemic effects of leaf hydroethanolic extracts

**DOI:** 10.3389/fphar.2025.1537071

**Published:** 2025-02-10

**Authors:** Arbi Guetat, Slimen Selmi, Abdelrhman T. Abdelwahab, Marwa A. Abdelfattah, Abd Ealrhman M. Elhaj, Roaa T. Mogharbel, M. J. A. Abualreish, Abdullah F. Alanazi, Karim Hosni, Naceur Mejri, Abdennacer Boulila

**Affiliations:** ^1^ Department of Biological Sciences, College of Sciences, Northern Border University, Arar, Saudi Arabia; ^2^ Laboratory of Functional Physiology and Valorization of Bioresources, Higher Institute of Biotechnology of Béja, Jendouba University, Béja, Tunisia; ^3^ College of Applied Medical Sciences, Northern Border University, Arar, Saudi Arabia; ^4^ Department of Chemistry, College of Sciences, Northern Border University, Arar, Saudi Arabia; ^5^ Laboratoire des Substances Naturelles, Institut National de Recherche et d’Analyse Physicochimique, Biotechpole de Sidi Thabet, Ariana, Tunisia; ^6^ Laboratory of Biotechnology and Nuclear Technology, National Center for Nuclear Science and Technology of Sidi Thabet, Ariana, Tunisia

**Keywords:** *Salvia deserti*, diabetes, lipid profile, phenolic compounds, antioxidant, antidiabetic affect, antihyperlipidemic effect

## Abstract

**Introduction:**

Diabetes is a major health issue that has reached alarming levels worldwide. Although their effectiveness, the antidiabetic drugs have many side effects such as cardiovascular diseases, kidney failure, and hepatic complications. Many plant species of the genus *Salvia* L. such as “Arabian desert sage” (*Salvia deserti*) should began to be the focus of phytochemical and bioactivity studies.

**Methods:**

The study aims to investigate, first-ever, the antidiabetic and antihyperlipidemic effects of the leaf hydroethanolic extract of *S. deserti,* an endemic and rare subshrub from Arabian desert. A single intraperitoneal injection of alloxan monohydrate (160 mg/kg) was used to induce type-2 diabetes. Development of diabetes was confirmed by measuring the blood glucose collected from the tail vein after 72 h of alloxan injection. Oral administration of hydroethanolic extracts of *S. deserti* at 200 and 400 mg/kg for 14 days were tested on the alloxan-induced diabetic mice and animals were divided into 6 groups of 6 mice each. The identity of phenolic compounds of the hydroethanolic extract of *S*. *deserti* was conducted through HPLC-Electrospray Ionization-Mass Spectrometry (LC-ESI-MS) analyses.

**Results:**

Oral administration of hydroethanolic extract of *S*. *deserti* at 200 and 400 mg/kg for 14 days significantly decreased blood glucose and restored the hepatic and renal function by reducing the levels of ALT, AST, ALP, GGT, LDH, urea and creatinine. An improved lipid profile as revealed by the reduced levels of TC, TG and LDL coupled with increased level of HDL was also observed. Moreover, treatment with *S. deserti* hydroethanolic extract relieved oxidative stress (reduction of MDA and H_2_O_2_) and the activity of antioxidant enzymes SOD, CAT and GPx. The presence of several phenolic compounds (derivatives of ferulic, coumaric, and caffeic acids, among other derivatives) could at least in part explain the obtained data and empower the use of *S. deserti* as a source of bioactive ingredients with antioxidant, antidiabetic and antihyperlipidemic properties.

**Discussion:**

Our research has unveiled *S. deserti* as a source of potential to effectively manage diabetes and its associated dyslipidemia by improving antioxidant status, recovery of the liver and kidney functions and presumably by increasing insulin secretion and sensitivity of peripheral tissues to insulin.

## 1 Introduction

Diabetes is a major health issue that has reached alarming levels worldwide. Today, approximately 537 million adults are living with diabetes and the total number is projected to rise and reach, by 2045, 783 million (one out of 8 adults) (IDF, 2021). The most common type of diabetes is type 2-diabetes which accounts for over 90% of all diabetes worldwide, and occurs when body became resistant to insulin (i.e., inability of body cell’s to respond fully to insulin) or when pancreatic beta cells failed to produce enough amounts of insulin (Hossain et al., 2024). These pathophysiological features and their subsequent disruption of glucose homeostasis leading to hyperglycemia. Type 2 diabetes was reported to be associated with macrovascular (heart, peripheral arterial and cerebrovascular diseases), microvascular (diabetic nephropathy, neuropathy and retinopathy) and other metabolic disorders including inflammation-induced atherosclerosis, dyslipidemia, hypertension, obesity, mitochondrial dysfunction and gut dysbiosis (Galicia-Garcia et al., 2020). The development of these pathological complications is usually influenced by genetic (ethnicity and family history, genetic predisposition), environmental (aging, obesity, sedentary lifestyle, unhealthy diet, low physical activity, smoking and alcohol intake and stress) and their interactions (Lu et al., 2020). Common treatments of diabetes namely, type 2- diabetes, involve a mechanism reversing hyperglycemia and include among others metformin and bromocriptine-QR (reduce insulin resistance in the peripheral tissues, improve hepatic insulin sensitivity and reduce hepatic glucose production); sulfonylurea and meglitinides (insulin secretagogues); pramlintide (reduces postprandial stimulated glucagon secretion); glucagon-like peptide-1 (enhances insulin secretion by β-cells and reduces glucagon secretion by the α-cells); and thiazolidinediones (reduces insulin resistance) (Ojo et al., 2023).

Although their effectiveness, some antidiabetic drugs have side effects like weight gain, osteoporosis, abdominal pain, cardiovascular diseases, kidney failure, and hepatic complications (Corathers et al., 2013). Therefore, the search for new antidiabetic agents with minimal side effects is a challenging task.

Epidemiological evidence showed that a lot of active phytochemicals isolated from different plant families are endowed with antidiabetic effect. For example, phenolic acids (e.g., chlorogenic, dicaffeoylquinic, ellagic acids), flavonoids (e.g., quercetin and its derivatives, naringin, myricetin, curcumin, resveratrol, pterostilbene); alkaloids (e.g., berberine, palmatine, canthin-6-one, trigonelline); saponins (e.g., gingenoside, diosgenin, oleanane); anthraquinones (e.g., emodin, catenarin); terpenes and terpenoids (e.g., β-caryophyllene, lupeol, oleanolic acid, β-amyrin, carosolic acid, maslinic acid); carotenoids (e.g., α-,β-carotene, zeaxanthin, lycopene, lutein); phytosterols (e.g., ergosterol, stigmasterol, lophenol, cycloartanol); polysaccharides (e.g., kinsenoside, α-arabinose, α-xylose, α-rhamnose) have manifest *in vitro* and *in vivo* hypoglycemic effects (Ardalani et al., 2021; Alam et al., 2022; Singh et al., 2022). The underlying mechanisms behind the antidiabetic activity of these phytochemicals include among others; i) enzyme inhibition (e.g., α-amylase, β-glucosidase, aldose reductase, dipeptidyl peptidase (DPP4), diacylglycerol-o-acyltransferase-1 (DGAT-1), fructose-1,6-biphosphate (F16P2), glycogen phosphorylase (GP); ii) boosting antioxidant status (i.e., activation of antioxidant enzymes like SOD, CAT, GPx, with a concomitant reduction of lipid peroxidation); iii) stimulation of insulin secretion by pancreas β-cells and its uptake by peripheral (e.g., muscular, adipose and hepatic) tissues reducing insulin resistance; iv) improving glucose tolerance (i.e., upregulation of the expression of insulin receptors, insulin receptor substrates, glycogene synthase kinase 3β, Akt serine/threonine kinase, etc.); and v) decreasing intestinal absorption by inhibiting sodium-dependent glucose transporter 1 (SGLT1) (Patel et al., 2012; Alam et al., 2022; Arifah et al., 2024; Przeor, 2022; Cherrada et al., 2024; Gawli and Bojja, 2024).

With approximately 1,000 species all over the world, the genus *Salvia abbottii* Urb. is one of the most important genera of the Lamiaceae family. It consists of annual, biannual and perennial herbaceous plants with woody subshrub (Uysal et al., 2023). Members of this genus are widely used for their aromatic, medicinal, cosmetic, ornamental, pharmaceutical and food-related applications. The plant of the genus are rich sources of essential oils and phenol-rich extracts with a wide array of biological activities including antioxidant, antimicrobial, enzymes-inhibitory, anti-inflammatory, wound healing, anticancer, anti-hyperglycemic, and neuroprotective properties (Ben Taârit et al., 2012; Jakovljević et al., 2019; Zhumaliyeva et al., 2023).

Most of the ethnopharmacological and phytochemical studies on *Salvia* are centered on species from the European and Asian clades (Ortiz-Mendoza et al., 2022).

Although their great diversity, limited *Salvia* species namely *S. officinalis, S. miltiorrhiza,* and *S. sclarea* were potentially exploited and extensively investigated, while many plant taxa of the genus *Salvia* have not received much attention. *Salvia deserti* Decne, an endemic species of the Egyptian and Arabian deserts (Will and Claßen-Bockhoff, 2014), is one of non-studied taxa of the genus *Salvia*. Excepting only one study focusing on volatile profiling of one Jordanian *S. deserti* specimen (Al-Jaber, 2017), data on the detailed phytochemical composition and bioactivities of this species are rather scarce. *S. deserti* is a subshrub and its native range is from Sinai, Palestine, Jordany and Saudi Arabia (POWO, 2024). It is a subshrub and grows primarily in the desert or dry shrubland biome. In the Northern Region of Saudi Arabia, the plant species is sparse and grows in some wadi tributaries during the rainy years. Therefore, the aim of the current study was to provide, for the first time, the phenolic profile and to evaluate the antidiabetic effects *in vivo* of a Saudi *S. deserti* specimen. Results of this study will not only extend our knowledge on the chemistry and bioactivity of this understudied plant species, but it also provides baseline information for eventual valorization of this rare species as a source of natural antidiabetic agents and/or for more nutraceutical purposes.

## 2 Materials and methods

### 2.1 Plant material collection and site of collection

In April 2024, *S. deserti* Decne. was collected from the wild. Plant material was sampled from a site situated about 45 km to the South of Arar city (Northern region of Saudi Arabia) in Wadi Al-Aqraa (30° 50′43″N; 40° 33′27″E). The botanical identification of the plant specimen was elaborated in the Department of Biology, College of Sciences, Northern Border University. A voucher specimen was deposited (SDr992) in the herbarium of the College of Sciences.

**FIGURE 1:**
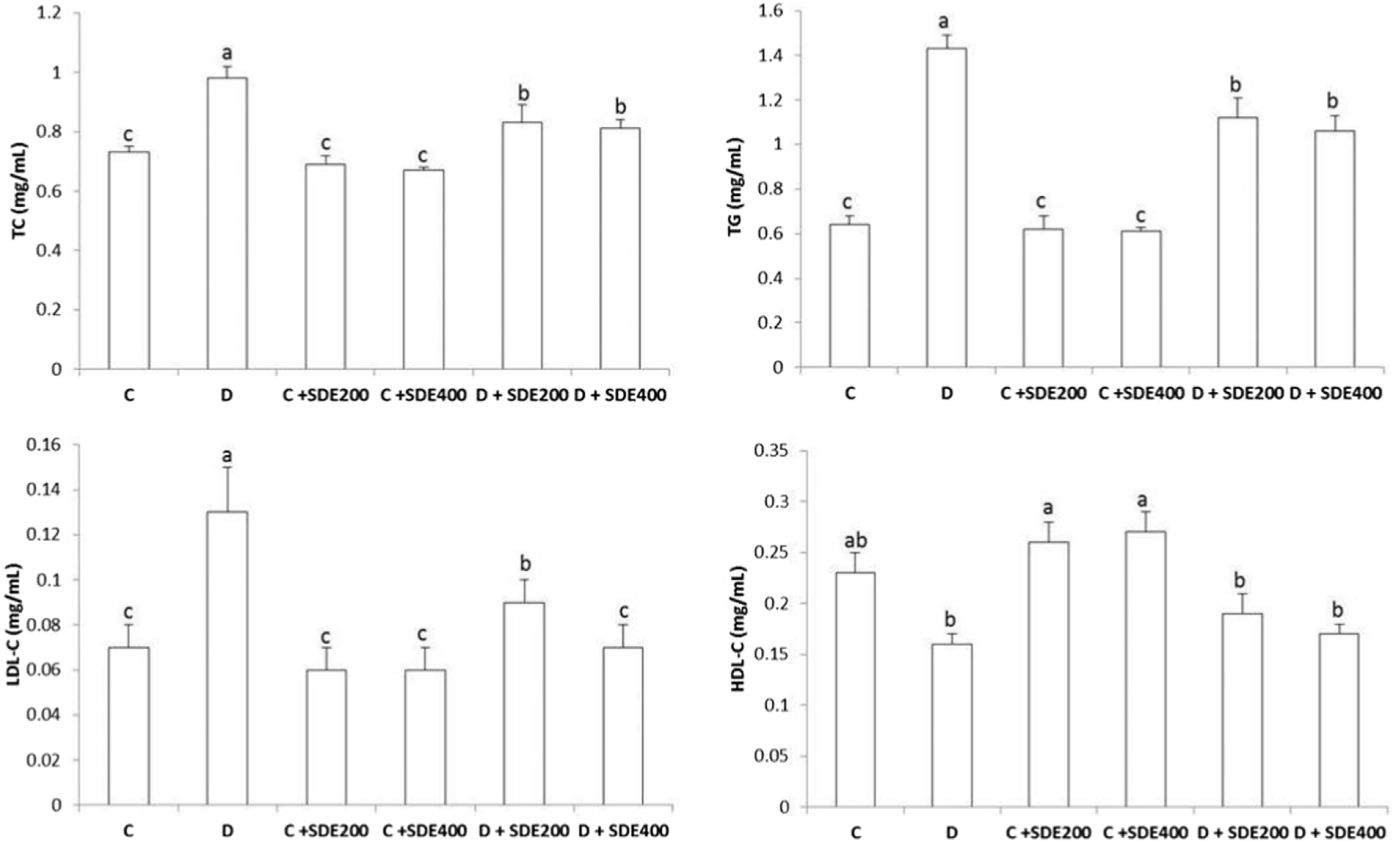
Effect of hydroethanolic extract on *Salvia deserti* on total cholesterol (TC), triglycerides (TG), LDL-cholesterol (LDL-C) and HDL-cholesterol (HDL-c) of different experimental groups. Values are given as mean ± SD (n = 6). Means with different superscript are statistically different at p < 0.05. C: Non diabetic control D: Diabetic control NDC + SDE_200_: Non diabetic control receiving hydroethanolic extract of *S. deserti* at a dose of 200 mg/kg NDC + SDE_400:_ Non diabetic control receiving hydroethanolic extract of *S. deserti* at a dose of 400 mg/kg D + SDE_200_: Diabetic animals receiving hydroethanolic extract of *S. deserti* at a dose of 200 mg/kg NDC + SDE_400:_ Diabetic animals receiving hydroethanolic extract of *S*. *deserti* at a dose of 400 mg/kg.

The altitude of the prospected site is situated 657 m above sea level. The region experiences average annual rainfall ranging from 159 to 182 mm (see Supplementary Material). This collection site falls within a Mediterranean desert continental climate, characterized as a dry zone with a hot, arid desert climate. The annual average temperature is 21.5°C, and precipitation is irregular, averaging 20.2 mm per year, predominantly occurring in the winter months, with January and December seeing the highest rainfall, often exceeding 90 mm. There are also several years with total annual rainfall below 100 mm. The habitat in Northern Saudi Arabia consists of a rocky desert, with hot, dry summers and cold winters, featuring only a few rainy days (see Supplementary data). According to the International Bioclimatic Classification System, the climate in this northern region is classified as a dry continental desert climate, positioned within the thermal range of the Mediterranean Sea (Figure 1, Supplementary Material). The soil in the Northern border region is underpinned by a calcareous plateau, averaging 530 m in height above sea level. The area features several non-permanent water flows due to fissures in the bedrock, and is locally referred to as the Al Widian Region, or the Zone of Many Wadies.

### 2.2 Extract preparation

The fresh aerial parts of the plant material were dried at room temperature in a shady place for 4 weeks. The air-dried powdered plant samples (10 g) were macerated on a shaker at room temperature for 24 h in 100 mL 70% ethanol. The obtained solution was filtered with 0.45 μm syringe filter and concentrated to dryness under reduced pressure in a rotary evaporator. The concentrated extract was freeze-dried and stored at −20°C until use.

### 2.3 Treatment and experimental design

Male Swiss albino rats (mean weight 30 ± 5 g) were obtained from the Society of Pharmaceutical Industries, Ben Arous, Tunisia. Mice were housed under standard conditions of temperature (22° ± 2°C), relative humidity (50%) and light (12 h light/dark cycle). They were fed a standard laboratory diet consisting of 23% protein, 12% moisture, 12% soybean meal, 10% fish meal, 10% wheat middling, 7% fiber, 3% fat, 3% corn gluten meal, 2.5% soybean oil, 1.5% molasses, 0.5% salt, minerals and vitamins (El Badr Utique-TN, Bizerte, Tunisia) and water *ad libitum.* All experiments were performed in accordance with the Care and Use of Laboratory Animals of the National Institute of Health (NIH publication No. 25-23) and approved by the ethical committee at the University of Monastir (Ethical approval: CER-SVS/ISBM 016/2021).

A single intraperitoneal injection of alloxan monohydrate (160 mg/kg) was used to induce type-2 diabetes. Development of diabetes was confirmed by measuring the blood glucose collected from the tail vein after 72 h of alloxan injection. Mice with blood glucose of 13 mM/L or higher were considered diabetic.

Animals were divided into 6 groups of 6 mice each. The *S. deserti* hydroethanolic extract (200 and 400 mg/kg) were orally administered by gavage once per day for 15 days.

Group 1: Normal control receiving basal diet
Group 2: Diabetic control receiving basal diet
Group 3: Normal control receiving basal diet and a dose of 200 mg/kg of *S. deserti* hydroethanolic extract
Group 4: Normal control receiving basal diet and a dose of 400 mg/kg of *S. deserti* hydroethanolic extract
Group 5: Diabetic mice receiving basal diet and a dose of 200 mg/kg of *S. deserti* hydroethanolic extract
Group 6: Diabetic mice receiving basal diet and a dose of 200 mg/kg of *S. deserti* hydroethanolic extract

At the end of experiment, mice were anesthetized using anesthetic ether and sacrificed by cervical dislocation. The post-mortem collection of blood was made using heparin-coated tubes and centrifuged at 3,000 × g for 10 min at 4°C. The serum was immediately collected for biochemical analyses.

Tissues from the liver and kidneys were dissected out and washed with ice-cold saline solution and then conserved at −80°C for enzymes and oxidative markers analysis.

### 2.4 Biochemical analyses

Serum glucose and the activity of aspartate transaminase (AST), alanine transaminase (ALT), alkaline phosphatase, lactate dehydrogenase (LDH) and gamma-glutamyl-transferase (GGT) as well as the levels of urea, uric acid, creatinine, C-reactive protein (CRP), triglycerides (TG), total cholesterol (TC), high-density lipoprotein-cholesterol (HDL-C) and low-density lipoprotein-cholesterol (LDL-C) were determined using commercial standard enzymatic colorimetric kits (Biomaghreb, Ariana, Tunisia).

For the determination of oxidative stress markers and the activity of endogenous antioxidant enzymes, liver and kidney tissues homogenates were prepared after homogenisation in 50 mM phosphate buffer saline (KH_2_PO_4_/K_2_HPO_4_; pH 7.4) followed by centrifugation at 10,000 × g for 10 min (4°C). The MDA content was determined according to the method of Draper and Hadley (1990) using the extinction coefficient of 1.56 × 10^5^ M^-1^ cm^-1^. The H_2_O_2_ content was estimated as described by Pick and Keisari (1981). Sulfhydryl group (-SH) was measured as described by Ellman (1959). Protein was estimated by the method of Bradford (1976) using bovine serum albumin as a standard.

The activity of superoxide dismutase (SOD) was measured spectrophotometrically at 480 nm as described by Misra and Fridovich (1972). Glutathione peroxidase (GPx) activity was determined at 412 nm using to the method of Flohé and Günzler (1984). Catalase (CAT) activity was assessed following the method of Abei (1984).

### 2.5 HPLC-ESI-MS analyses

LC-DAD-ELSD-ESI/MS analyses was carried out on an Agilent 1,200 series HPLC (Courtaboeuf, France) equipped with ELSD and DAD detectors and coupled to an Electrospray-Ionization Mass Spectrometer (Thermo Fisher Scientific, San Jose, CA, United States). The ELSD was used under the following conditions: temperature: 40°C; nebulizing gas pressure: 3.6 bar; gain: 1; sampling time: 100–10 Hz; filter: 3 s. The DAD provided two characteristic UV wavelengths: 254 and 280 nm. Separation of compounds was performed on a Luna 5 µm C_18_ column (250 mm × 4.6 mm i. d.) from Phenomenex (Le Pecq, Ile-deFrance, France).

Mobile phase consisted in a multistep gradient of water (A) and acetonitrile (B), both acidified with 0.1% formic acid. The flow rate was 0.3 mL/min. The gradient was: 0–15 min, 50% B; 15–20 min, 50% B; 20–30 min, 50%–75% B; 30–35 min, 75% B; 35–40 min, 75%–100% B; 40–45 min, 100% B; 45–48 min, 100%–10% B; 48–55 min, final isocratic step at 10% B.

The detection using ESI was performed both in positive and negative modes with the following parameters: capillary temperature: 275°C; capillary voltage: ±3.8 and 4 kV; cone voltage: ±10 V. Nitrogen was used as nebulization gas with a cone flow rate of 50 L/h and a solvation flow rate of 10 L/h. The scanning range was m/z 100 to 1,200.

### 2.6 Statistical analyses

The one-way analysis of variance (ANOVA) followed by Tukey’s post-hoc test for multiple comparisons was used to detect inter-group differences. All analyses were performed using the Statistical Package for the Social Sciences (SPSS) software (version 18.0 for Windows, SPSS Inc., Chicago, IL, United States). Results at *p* < 0.05 were considered statistically significant.

## 3 Results and discussion

### 3.1 Effect of the hydroethanolic extract of *S. deserti* on blood glucose, hepatic and renal functions and lipid profile

Table 1 summarizes the levels of blood glucose and serum biochemical markers in normal, alloxan-induced diabetic and treated-diabetic animals with *S. deserti* hydroethanolic extracts. As shown, the intraperitoneal injection of a single dose of alloxan monohydrate induced a diabetes mellitus in mice as it raised significantly the levels of blood fasting glucose following the ROS-induced necrosis of pancreatic β-cells resulting ultimately to hyperglycemia. This pathogenesis of hyperglycemia results from deficient insulin production and a subsequent release of hepatic glucose versus impaired glucose utilization by peripheral tissues (Cryer, 2012).

**TABLE 1:** Effects of hydroethanolic extracts of *S. deserti* on blood glucose and hepatic, and renal function parameters.

Parameters	NDC	D	NDC + SDE_200_	NDC + SDE_400_	D + SDE_200_	D + SDE_400_
Glucose (mM)	6.54^d^ ± 0.28	15.63^a^ ± 0.90	6.78^d^ ± 0.37	6.38^d^ ± 0.19	11.33^b^ ± 0.80	9.09^c^ ± 0.63
ALT (UI/L)	90^e^ ± 0.71	250^a^ ± 7.54	102^d^ ± 3.18	118^c^ ± 5.26	189^b^ ± 8.66	171^b^ ± 5.67
AST (UI/L)	50^e^ ± 2.12	172^a^ ± 3.18	61^d^ ± 2.7	52^e^ ± 2.46	124^b^ ± 3.54	109^c^ ± 4.06
GGT (UI/L)	30^d^ ± 2.83	84^a^ ± 4.13	32^d^ ± 1.14	32^d^ ± 3.43	64^b^ ± 3.54	57^c^ ± 2.08
ALP (UI/L)	60^d^ ± 3.41	289^a^ ± 8.59	64^d^ ± 2.88	61^d^ ± 4.23	118^b^ ± 5.81	103^c^ ± 6.69
LDH (UI/L)	624^c^ ± 27	1219^a^ ± 23	615^c^ ± 38	648^c^ ± 42	934^b^ ± 49	906^b^ ± 53
CRP (mg/L)	1.8^e^ ± 0.07	8.6^a^ ± 0.16	2.3^d^ ± 0.09	1.92^e^ ± 0.07	6.09^b^ ± 0.11	5.38^c^ ± 0.09
Urea (mmol/L)	7.98^d^ ± 0.83	12.39^a^ ± 0.42	8.36^c^ ± 0.43	7.91^d^± 0.43	11.14^a^ ± 0.96	10.83^b^ ± 0.88
Uric acid (mmol/L)	0.36^a^ ± 0.02	0.14^d^ ± 0.01	0.33^a^ ± 0.01	0.34^a^ ± 0.02	0.23^c^ ± 0.01	0.27^b^ ± 0.02
Creatinine (μmol/L)	121^c^ ± 5.8	193^a^ ± 7.3	119^c^ ± 6.8	114^c^ ± 4.6	158^b^ ± 6.3	156^b^ ± 7.4

*Values are given as mean ± SD (n = 6). Means with different superscript are statistically different at *p* < 0.05.

C, non diabetic control.

D, diabetic control.

NDC + SDE_200_: Non diabetic control receiving hydroethanolic extract of *S. deserti* at a dose of 200 mg/kg.

NDC + SDE_400:_ Non diabetic control receiving hydroethanolic extract of *S. deserti* at a dose of 400 mg/kg.

D + SDE_200_: Diabetic animals receiving hydroethanolic extract of *S. deserti* at a dose of 200 mg/kg.

NDC + SDE_400:_ Diabetic animals receiving hydroethanolic extract of *S*. *deserti* at a dose of 400 mg/kg.

Hyperglycemia was also associated with a drastic alteration of the hepatic parameters (alanine aminotransferase (ALT), aspartate aminotransferase (AST), alkaline phosphatase (ALP) and gamma-glutamyltransferase (GGT)) and renal (urea, uric acid and creatinine)) functions. Such manifestations were described as the main hallmarks of type-2 diabetes in alloxanized diabetic mice’s (Alam et al., 2014). Interestingly, in addition to insulin resistance reflected by the increased level of GGT (marker of oxidative stress and insulin resistance), diabetic mice experienced inflammation as shown by the increased level of C-reactive protein (CRP).

Administration of 70% ethanolic extract of *S. deserti* dose-dependently restored glycemic homeostasis indicating its ability to manage alloxan-induced hyperglycemia and counteract glucotoxicity. The hypoglycemic effects of the hydroethanolic extract may be due to the improvement of insulin output by intact and/or regenerated β-cells of the islets of Langherhans, or due to the presence of active components with insulin-mimetic effects that increase glucose uptake by peripheral tissues (muscle and adipose tissues). However, as the glucose level remains unaltered in normal mice receiving hydroethanolic extracts of *S. derserti*, it is plausible to suggest that the observed hypoglycemic action was attributed to its insulin stimulatory effect rather than insulin-mimetic action. Moreover, an antioxidant-mediated hypoglycemic effect owing to the presence of putative antioxidants and/or its ability to potentiate the endogenous enzymatic antioxidant system could also be hypothesized as another mechanism of the hypoglycemic action of the hydroethanolic extract of *S. derserti* (Mejri et al., 2018).

Administered at a dose of 200 and 400 mg/kg, the hydroethanolic extract exerted a protective effect against glucotoxicity-induced liver injury as it normalized the levels of AST, ALT, ALP and GGT. In addition to its normoglycemic effect, the protective action of *S. deserti* extract could be related to the reduced lipid accumulation in liver and its subsequent free fatty acid-induced hepatotoxicity (Guo et al., 2017). A surrogate and/or complementary mechanism involving an increased antioxidant response (as revealed by the normalization of the activity of the GGT that is implicated in the synthesis of the water-soluble antioxidant glutathione (GSH)) could also be responsible for the observed hepatoprotective effect (Javid et al., 2023). Concomitantly, the reduction of GGT could be indicative to a reduced insulin resistance explaining thus the hypoglycemic effect of the hydroethanolic extract of *S. deserti*.

The ability to protect kidney from hyperglycemia-induced nephropathy was also recorded for the hydroethanolic extract of *S. deserti* as it improved all renal parameters (creatinine, uric acid, and urea). Such beneficial effects on kidney function could be linked to its ability to alleviate oxidative stress and inflammatory markers (as revealed by the reduced level of CRP in diabetic animals treated with hydroethanolic extract) (Damera et al., 2011).

### 3.2 Effect of the hydroethanolic extract of *S. deserti* on lipid profile

Dyslipidemia is a major complication of type 2 diabetes and is associated with increased levels of the classical lipid markers TG, TC and LDL (Figure 1). These parameters are repeatedly considered as major risk factors for coronary heart disease and chronic kidney disease (Lanktree et al., 2018; Cheung et al., 2023). Regardless of the dose (200 or 400 mg/kg), supplementing *S. deserti* hydroethanolic extract significantly reduced all these biomarkers compared to diabetic animals, indicating thus its lipid lowering property. Despite that the mechanism of action is unclear; the observed lipid lowering effect could be attributed to its ability to inhibit the activity of different enzymes involved in the metabolism of cholesterol, TG and VLDL such as glucose-6- phosphate dehydrogenase (G6PD), 3-hydroxy-3-methylglutaryl coenzyme A (HMG-CoA) reductase, Acyl coenzyme A: cholesterol acyltransferase (ACAT) and paraoxonase (PON), among others (Mollazadeh et al., 2019; Alyahya et al., 2023).

Remarkably, a significant increase in the level of HDL (beneficial cholesterol) was observed in the non-diabetic control animals suggesting the clearance ability of cholesterol from blood of *S. deserti* extracts which, in turn, could be associated with low incidence of cardiovascular disease and beneficial effects on endothelial physiology owing to its atheroprotective, anti-inflammatory, anti-thrombotic and antioxidant properties (Pirillo et al., 2019). In contrast, reciprocal trends (low HDL levels versus elevated cholesterol levels) were observed in diabetic control and to a lesser extent in diabetic animals treated with hydroethanolic extract of *S. deserti*. The observed loss of cholesterol efflux might presumably be due to hyperglycemia-induced HDL dysfunction and impaired insulin sensitivity of adipose tissue and liver (Xepapadaki et al., 2021; Martagon et al., 2024). Alterations of the structure and function of HDL (*i.e.*, abnormalities in number, size, composition and function of HDL particles) could also be associated with inflammation (increased levels of CRP) and altered antioxidant status of diabetic animals. The slight improvement of HDL functionality (reduction of total cholesterol) in diabetic animals treated with the hydroethanolic extract of *S. deserti* reflects an improved HDL functionality as their levels were similar to the control diabetic animal. This may be associated with the ability of *S. deserti* to mitigate the hyperglycemia-induced oxidative stress and to boost the endogenous antioxidant systems. Alleviating oxidative stress may, in turn, reduce the oxidation of LDL resulting thus in a manifest amelioration of the lipid profile. To verify this assumption, oxidative markers and the activity of antioxidant enzymes were determined.

### 3.3 Effect of the hydroethanolic extract of *S. deserti* on oxidative stress markers and the activity of endogenous antioxidant enzymes

**FIGURE 2:**
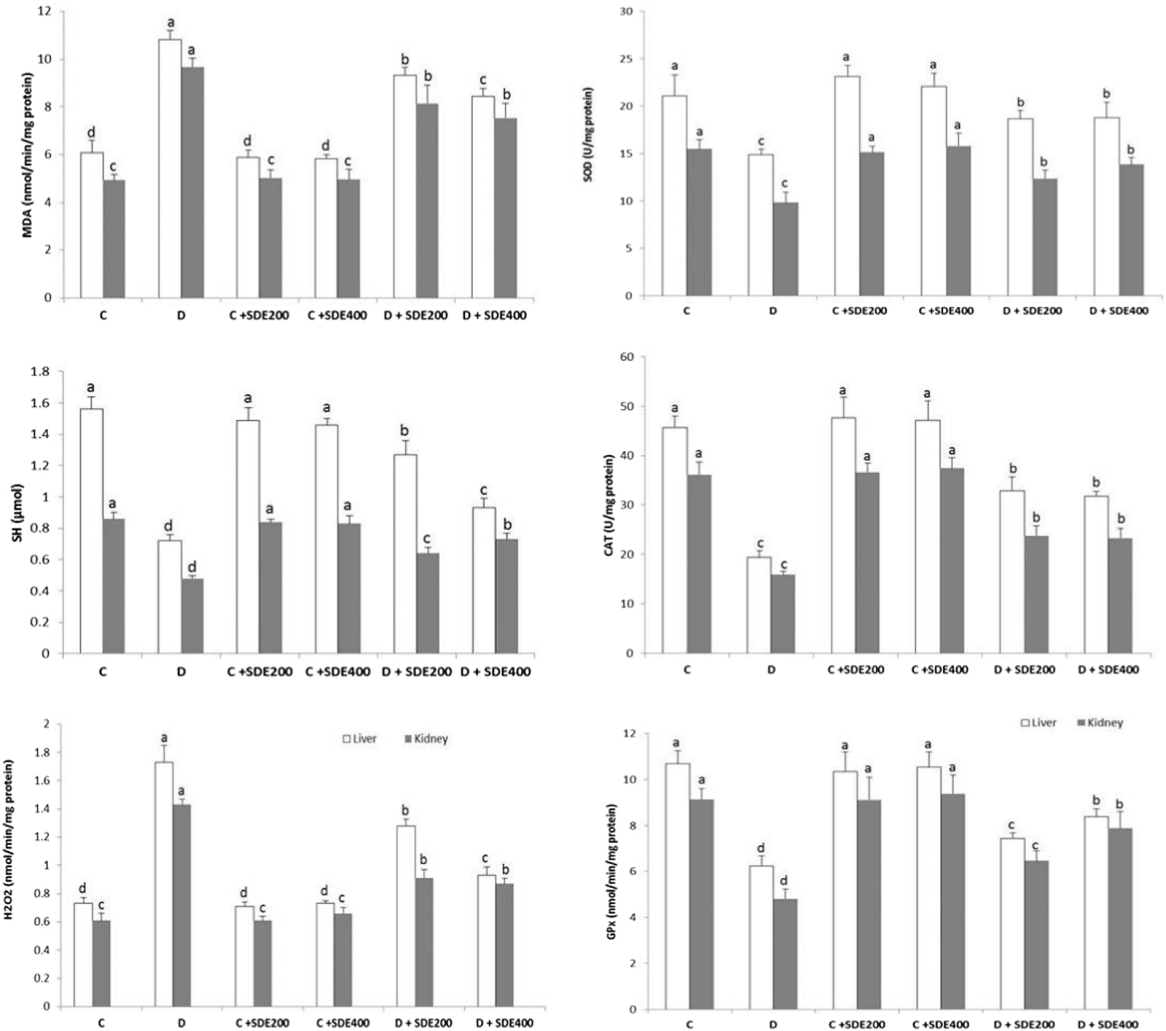
Effects of hydroethanolic extract of *S. deserti* on oxidative stress markers (MDA, H_2_O_2_ and (-SH) group) and the activity of antioxidant enzymes (SOD, CAT and GPx) on different experimental groups. Values are given as mean ± SD (n = 6). Means with different superscript are statistically different at p < 0.05. C: Non diabetic control D: Diabetic control NDC + SDE_200_: Non diabetic control receiving hydroethanolic extract of *S. deserti* at a dose of 200 mg/kg NDC + SDE_400:_ Non diabetic control receiving hydroethanolic extract of *S. deserti* at a dose of 400 mg/kg D + SDE_200_: Diabetic animals receiving hydroethanolic extract of *S. deserti* at a dose of 200 mg/kg NDC + SDE_400:_ Diabetic animals receiving hydroethanolic extract of *S*. *deserti* at a dose of 400 mg/kg.

The extent of lipid peroxidation (determined as malondialdehyde (MDA) content), H_2_O_2_ and thiol (-SH) groups, and the activity of the antioxidant enzymes SOD, CAT and GPx were determined in liver and kidney (Figure 2). As shown, diabetic animals experienced severe oxidative stress as their liver and kidney tissue homogenates exhibited a significantly (*p* < 0.05) higher amount of H_2_O_2_ and MDA *versus* a reduction of thiol content. Concomitantly, the observed state of oxidative stress was exacerbated by the depletion of the activity of the antioxidant enzymes SOD, CAT and GPx. The impairment of the activity of endogenous antioxidant enzymes was primarily due to their inactivation by the excess of H_2_O_2_ or by its glycosylation (Dewanjee et al., 2009). The oxidative stress-induced lipid peroxidation represents the main causative factors for the physiopathology of diabetes like insulin resistance, impaired insulin secretion and glucose homeostasis, production of inflammatory mediators, and perturbation of lipid profile (Zhao et al., 2011).

All these parameters were partially restored after oral administration of the hydroethanolic extract of *S. deserti*. Mitigation of the hyperglycemia-induced oxidative stress was presumably mediated through a direct quenching of free radicals; preventing their formation and/or boosting the activity of the endogenous antioxidant enzymes by inhibiting their peroxidation and glycation and/or inducing their protein synthesis (Al-Azzawie and Alhamdani, 2006). The enhanced SOD activity may, in turn, prevent H_2_O_2_-induced inactivation of CAT and GPx.

The presence of putative antioxidants in the hydroethanolic extract of *S. deserti* may be responsible for relieving diabetes-induced oxidative stress. To shed more light on this aspect, a HPLC-ESI-MS profiling of the phenolic components was carried out.

### 3.4 HPLC-ESI-MS of the hydroethanolic extract of *S. deserti*

**FIGURE 3:**
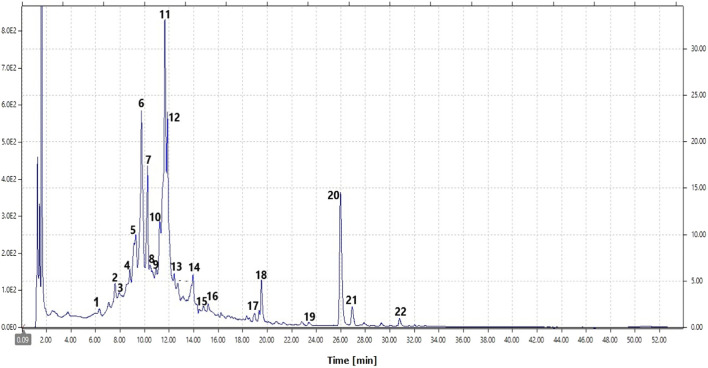
Typical HPLC-DAD chromatogram of the hydoethanolic extract of *S. deserti* (peak assignments are given in Table 2).

The typical chromatogram of the hydroethanolic extract of *S. deserti* is shown in Figure 3. The identity of phenolic compounds along with the retention time, molecular weight, and molecular ion in positive and negative ion mode is depicted in Table 2. Identified compounds included ferulic acid derivatives, coumaric acid derivatives, caffeic acid derivative (yunnaneic acid D, rosmarinic acid and its methyl derivative), the biflavone ginkgetin, flavone (chrysin-6-C-arabinoside-8-C-glucoside, genkwanin, luteolin-*O*-glucuronide, luteolin acetylglucoside), flavonols (kaempferol, isorhamnetin-*O*-hexoside), the xanthone mangiferin and a fatty acid derivative trihydroxylinoleic acid.

**TABLE 2:** Phenolic compounds identified from leaf hydroethanolic extracts of *Salvia deserti*.

Peak number	*Rt* (min)	M.W.	Molecular ion [M-H]-	Molecular ion [M + H]+	Identification	References
1	6.54	326	325	-	Feruloyl arabinose isomer	Zimmermann et al. (2011)
2	7.81	326	325	-	Feruloyl arabinose isomer	Zimmermann et al. (2011)
3	8.04	338	-	339	Coumaroyl quinate	Serrano et al. (2023)
4	8.72	368	-	369	Feruloyl quinate	Wianowska and Gil (2019)
5	9.11	294	295	-	*p*-Coumaric acid pentoside	Afonso et al. (2019)
6	9.80	540	539	-	Yunnaneic acid D	Afonso et al. (2019)
7	10.25	566	-	567	Ginkgetin	Nasr et al. (2023)
8	10.39	310	309	-	Feruloyl-malic acid isomer I	Mekam et al. (2019)
9	11.01	310	309	-	Feruloyl-malic acid isomer II	Mekam et al. (2019)
10	11.39	340	-	341	Sinapoyl malate	Oszmiański et al. (2013)
11	11.75	360	359	-	Rosmarinic acid	Boulila et al. (2015)
12	11.96	182	-	183	Syringaldehyde	Xu et al. (2024)
13	12.23	548	547	549	Chrysin-6-*C*-arabinoside-8-*C*-glucoside	Zengin et al. (2019)
14	13.88	374	373	-	Methyl rosmarinic acid	Zeng et al. (2006)
15	14.75	286	285	287	Kaempferol	Abdennacer et al. (2015)
16	15.10	344	343	345	Danshensu-rhamnoside	Zeng et al. (2006)
17	19.36	478	477	-	Isorhamnetin-*O*-hexoside	Yang et al. (2012)
18	19.43	284	283	285	Genkwanin	Mena et al. (2016)
19	23.25	422	421	423	Mangiferin	Yehia and Altwaim (2023)
20	25.85	462	461	-	Luteolin-*O*-glucuronide	Serrano et al. (2023)
21	26.79	490	489	-	Luteolin acetylglucoside	Almanza et al. (1997)
22	30.84	328	-	329	Trihydroxylinoleic acid	Serrano et al. (2023)

In addition to their chemotaxonomic significance as chemotaxonomic markers of the genus *Salvia,* yunnaneic acid D and danshensu-rhamnoside are well-known for their free radical scavenging activity and lipid peroxidation inhibitory effect (Lu et al., 2020) which could at least in part contribute to the antioxidant, antidiabetic and antihyperlipidemic effect of *S. deserti*. Other components such as feruloyl arabinose (Fang et al., 2013), sinapoyl malate (Nićiforivić and Abarmovič, 2014), genkgetin (Adnan et al., 2020), luteolin-7-*O*-glucuronide (Cho et al., 2020), isorhamnetin-*O*-hexoside (Farag et al., 2020; Wang et al., 2023), syringaldehyde (Wu et al., 2022), rosmarinic acid (Silva et al., 2022), genkwanin (El Menyiy et al., 2023), and mangiferin (Mistry et al., 2023), among others could also be responsible for such activities.

### 3.5 Conclusion

Results of the present study highlight that *S. deserti* hold potential to effectively manage diabetes and its associated dyslipidemia by improving antioxidant status, recovery of the liver and kidney functions and presumably by increasing insulin secretion and sensitivity of peripheral tissues to insulin. The presence of bioactive phenolic acid, flavonoids and xanthone with recognized antioxidant and antihyperglycemic and antihyperlipidemic activities could explain the species bioactivity and encourage its use for pharmaceutical and ethnopharmacological applications.

## Data Availability

The raw data supporting the conclusions of this article will be made available by the authors upon request.
