# Supplementary material for: Salvia deserti Decne., an endemic and rare subshrub from Arabian desert: antidiabetic and antihyperlipidemic effects of leaf hydroethanolic extracts
Source: Front Pharmacol. 2025 Feb 10;16:1537071. doi: 10.3389/fphar.2025.1537071 (PMC11847675; doi:10.3389/fphar.2025.1537071)
Supplement: Supplementary file 1 [file DataSheet1.doc]

**Supplementary materials**

**Site of plant collection: Ţalaat ‘Ammār**


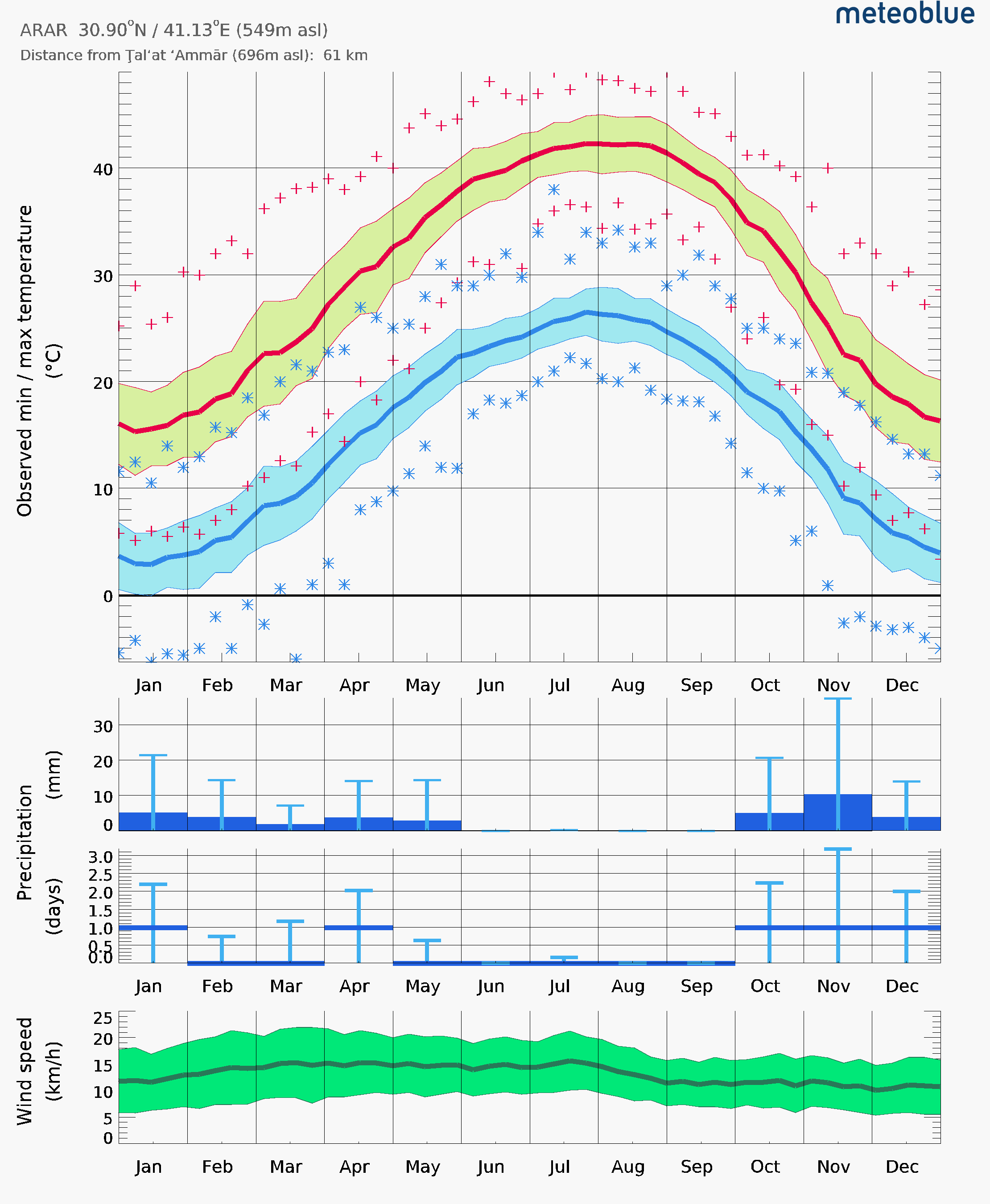


**Observed climate data for Ţalaat ‘Ammār (site situated about 15 Km from Wadi Al-Aqraa)**


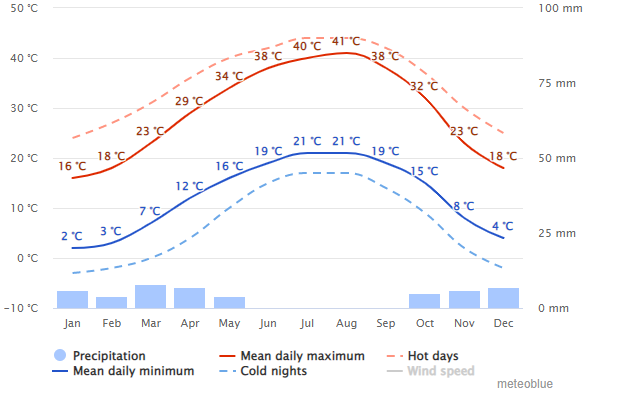


**Modelled climate data for Ţalaat ‘Ammār (Population 12)**

| **Years** | **Precipitation (mm)** |
| --- | --- |
| **2018** | 260.1 |
| **2019** | 266.051 |
| **2020** | 43.635 |
| **2021** | 50.519 |
| **2022** | 127.45 |

**AP = Annual precipitation (mm/yr) for Ţalaat ‘Ammār (site situated about 15 Km from Wadi Al-Aqraa)**

**Source**: <https://guillermo-ortega.users.earthengine.app/view/agroclim-scanner>


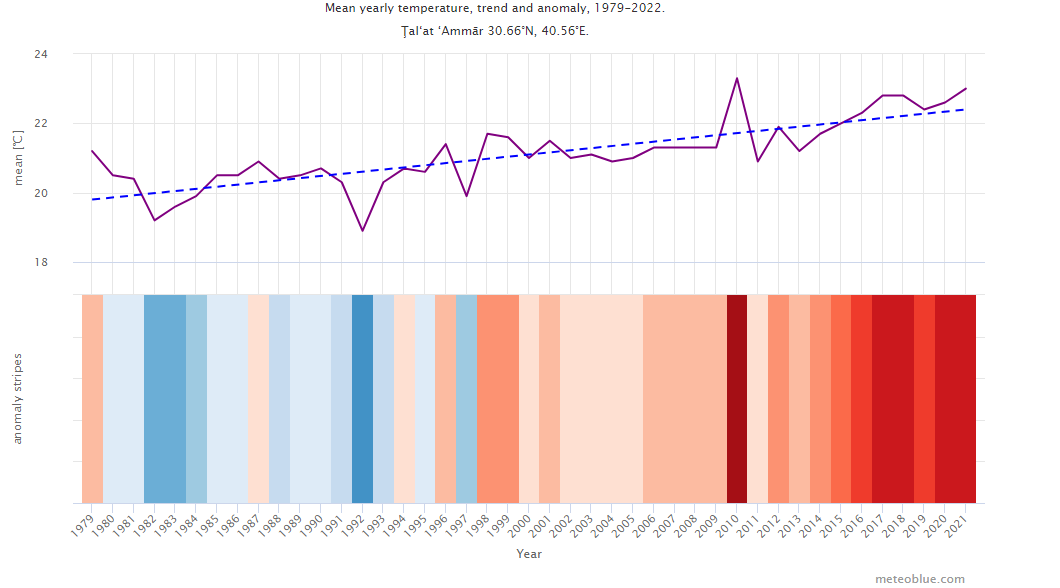


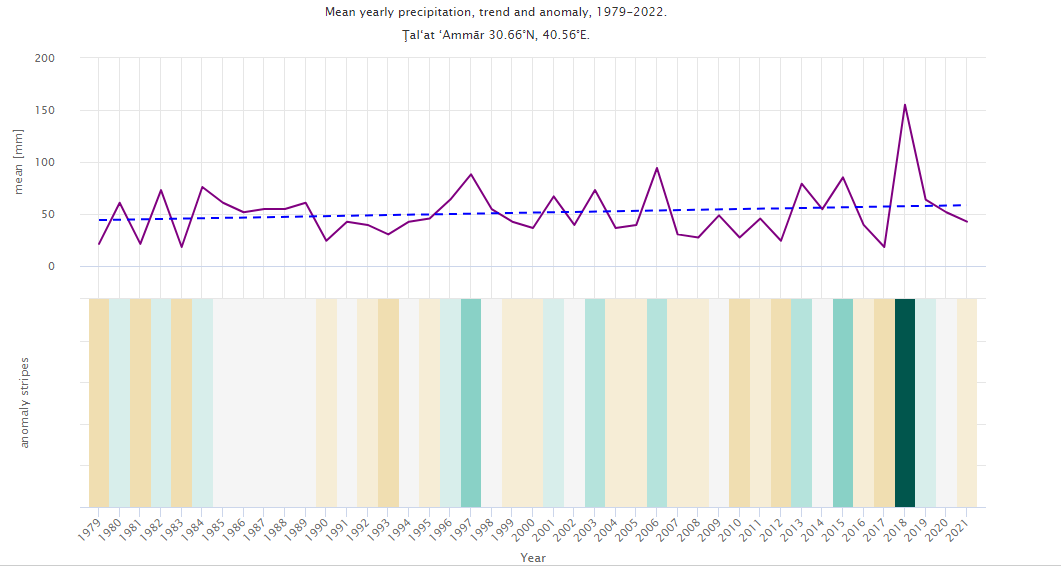


**Mean Yearly temperature and precipitation for Ţalaat ‘Ammār (Population 12): Trends and anomalies**
